# Fabrication of Ring-Shaped Deposits of Polystyrene Microparticles Driven by Thermocapillary Mechanism

**DOI:** 10.3390/ma14185267

**Published:** 2021-09-13

**Authors:** Mohammed Al-Muzaiqer, Natalia Ivanova, Denis Klyuev

**Affiliations:** 1 Photonics and Microfluidics Laboratory, X-BIO Institute, University of Tyumen, 6 Volodarskogo, 625003 Tyumen, Russia; m.al-muzajker@utmn.ru (M.A.-M.); d.s.klyuev@utmn.ru (D.K.); 2 Microfiltration Processes Laboratory, WCRC “Advanced Digital Technologies” University of Tyumen, 6 Volodarskogo, 625003 Tyumen, Russia

**Keywords:** spherical polystyrene microparticles, self-assembled materials, microparticle deposits, thermocapillary flows, evaporative lithography, heat and mass transfer

## Abstract

Fabrication of ring-shaped deposits of microparticles on solid surfaces with the desired length scales and morphology of particle arrangements is of great importance when developing modern optical and electronic resonators, chemical sensors, touch screens, field-emission displays, porous materials, and coatings with various functional properties. However, the controlled formation of ring-shaped patterns scaling from a few millimeters up to centimeters with simultaneous control of particle arrangement at the microscale is one of the most challenging problems in advanced materials science and technology. Here, we report a fabrication approach for ring-shaped structures of microparticles on a glass surface that relied on a local thermal impact produced by the subsurface heater and heat sink. Thermocapillary convection in the liquid covering microparticles in combination with evaporative lithography is responsible for the particle transport and the assembling into the ring-shaped patterns. An advantageous feature of this approach is based on the control of thermocapillary flow direction, achieved by changing the sign of the temperature gradient in the liquid, switching between heating and cooling modes. That allows for changing the particle transfer direction to create the ring-shaped deposits and dynamically tune their size and density distribution. We have studied the influence of the power applied to the heat source/sink and the duration of the applied thermal field on the rate of the ring fabrication, the sizes of the ring and the profile of the particle distribution in the ring. The proposed method is flexible to control simultaneously the centimeter scale and microscale processes of transfer and arrangements of particles and can be applied to the fabrication of ring structures of particles of different nature and shape.

## 1. Introduction

Multifunctional materials, solid surfaces and films with extraordinary properties are of great importance in advanced material science, modern industry, and medical diagnostics. One of the approaches to the design of such materials is based on the deposition of 2D and 3D structures, and planar patterns, on the surfaces of interest using nano- and microparticles of arbitrary shapes, having specific physical, chemical, and biological properties. For the implementation of that approach, the technology referred to as the evaporative-induced self-assembly is utilized [1]. This includes the interaction between the physical mechanisms arising in the course of the spontaneous evaporation of droplets, films and meniscus of colloidal solutions, upon various external (pre-defined) passive factors, and external forces’ impacts on that system in combination with the evaporation process. As an example, a spontaneous evaporation of a sessile colloidal droplet leads to the accumulation of particles at the droplet edge and the formation of a ring-shaped pattern. This effect is known as the coffee ring effect [2]. The intense evaporation at the droplet edge induces in a bulk solution the radially outward-directed compensatory flow resulting in the ring-like deposit at the droplet periphery. If the substrate with the sessile colloidal droplet is heated, then the ring-like pattern will be modified into an eye-like pattern consisting of a large central stain and a thin ring [3]. In this case, the thermal Marangoni flow (thermocapillary flow) arising from a temperature gradient between the droplet edge and the top brings particles to the central area. The inward-directed radially thermocapillary flow dominates the outward-directed compensatory flow preventing the ring formation until the droplet becomes thin enough to decelerate thermocapillary flow and allow a thin ring to be deposited. However, another way is that of applying a local small jet of alcohol vapor to a free portion of the surface of the evaporative colloidal droplet [4]. This causes the inward-directed radially Marangoni flow along the droplet surface due to a concentration gradient between the apex and the edge of the droplet. Similar to the previous case, Marangoni flow transfers particles to the center of the droplet base and forms a local deposit in the center. Changes in the position of the small vapor jet allow flexible control of the deposit shape and configuration.

A compact monolayer or a mountain-like deposit can be obtained on a solid surface via spontaneous evaporation of a sessile droplet containing micron size particles [5,6]. In this case, the mechanism of particle transfer relies on an immersion capillary force, which affects large particles at the liquid–air interface. Using a thermocapillary mechanism caused by local heating of a thin evaporative liquid layer is another method to create a circular shape monolayer or multilayer depositions of particles with sizes of tens of microns [7]. Thus, the evaporation-induced self-assembling technology enables the creation of materials for medical diagnostics [8,9,10], modern materials for photonics optoelectronics and optics [11,12,13,14,15,16], transparent conductive coatings for flexible films [17,18], bioinspired hierarchical materials and surfaces with various functional properties [19,20], as well as a wide range of materials for other different purposes [21,22,23,24,25,26].

Despite significant advances in the production of coatings and materials with required properties, there is still a challenge in creating patterns of a desired geometry on sub-millimeter spatial scales when the dimensions of the structures are orders of magnitude larger than the sizes of the particles. In particular, the fabrication of the ring-shaped deposits is of great demand in such applications as optical and electronic resonators, touch screens, displays, biochemical analysis, and many others [10,17,18,22].

Here we demonstrate an effective method for the creation of ring-shaped deposits of microparticles on solid surfaces, which enables control of the ring dimensions on the plane and the particle arrangement along the width of the ring. The method is based on a combination of thermocapillary convective flow in the liquid, covering microparticles, on the one hand, and evaporative lithography on the other hand [7]. A key feature of the developed method is based on the control of the particles transfer direction through the switching between heating and cooling modes.

## 2. Materials and Methods

### 2.1. Experimental Procedure

**Figure 1:**
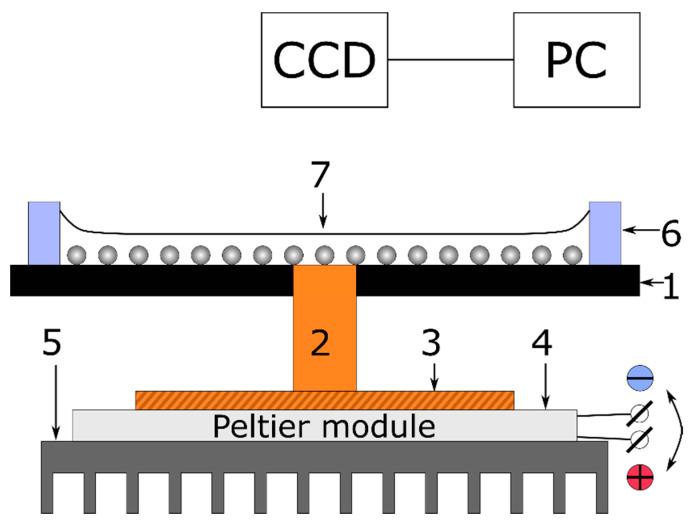
Sketch of the experimental setup for studying the formation of the ring-shaped deposit: 1—glass substrate, 2—copper rod, 3—copper plate, 4—Peltier module, 5—aluminum radiator, 6—polymeric ring, wall of the fluidic cell, 7—polystyrene particles on the substrate covered with a liquid layer.

Polystyrene microspheres (mean diameter d= 50 µm, material density $\rho_{p}=$ 1060 kg/m^3^) were purchased from LenChrom (Saint Petersburg, Russia). Isopropanol ($\rho_{l}≅$ 785 kg/m^3^, surface tension γ= 21.74 mN/m, thermocapillary coefficient $\gamma_{T}^{\prime }=$ 0.0789 mN/(m K)) were purchased from Sigma-Aldrich (Moscow, Russia) and used as a carrier liquid. As a solid substrate, a black welding glass (12DIN, in size of 44 × 35 × 3 mm^3^) was used. A copper rod of 1.8 ± 0.1 mm in diameter was hermetically embedded and flush with the surface of the glass substrate, through a hole drilled in the center of the substrate. The rod was thermally glued to a Peltier module (TEC-30-32-127; 33.4 W; 30 × 30 × 3.2 mm^3^), which was connected to an aluminum heat radiator (Figure 1). The copper rod served as a local heater or cooler (heat sink) depending on the polarity of the voltage applied to the Peltier module, which allowed to increase or decrease the temperature of the surface relative to the room temperature, $T_{0}$. To form a fluidic cell, a polymeric ring of $R_{0}=$ 10 mm in radius was glued to the welding glass substrate (Figure 1). Experiments were carried out under the following initial conditions: Thickness of the layer h≅ 200 µm, and number of particles n≅ 43 × 10^3^. The latter was roughly estimated using formula $n=6m/\pi d^{3}\rho_{p}$, where m= 3 mg is the mass of particles weighed with a precision balance (Ohaus Adventurer AX124, resolution 0.0001 g). The particles were deposited onto the initially dry glass substrate. Then, the required volume of isopropanol was added into the cell to set the layer thickness.

Experiments on the ring-shaped deposit formation were carried out according to the following protocol. In the beginning, the Peltier module was connected to the power supply in opposite polarity, and as a result, the temperature of the rod was lowered for a specified time span; then, the polarity of applied voltage was changed and the rod started to serve as a heater, increasing the temperature. After the ring-shaped deposit was formed, the system was left untouched until isopropanol was completely evaporated. Experiments were performed with the cooling time spans, τ, varying from 5 to 50 s and with various values of the cooling power of the Peltier module, ranging between 1.2 and 32 W (electrical power). Experiments were repeated 5 times for each value of power and cooling time span.

The experiments were recorded using a microscope Axio Zoom V16 with the lens Zeissapoz 1.5x/0.37 FWD 30 mm equipped with the CCD camera Zeiss Axiocam 506 color (Carl Zeiss Microscopy GmbH, Jena, Germany). The captured images were analyzed using the method proposed in our previous study [7]. In particular, the evolution of the area cleared from particles $S_{in}\left(t\right)$, which is the inner area of the ring-shaped deposit, and the area of the final ring-shaped deposit, $S_{r}$, were measured, in dependence on the cooling time spans and the cooling power of the Peltier module. The evolution and radial distributions of temperature were measured with an IR camera (Flir A655sc, spectral range 7.5–14 µm, ±2 °C). The morphology of the particle arrangement (distribution) across the width of the ring-shaped deposit was characterized using a scanning electron microscope (TESCAN Mira 3 LMU, Brno-Kohoutovice, Czech Republic) with SE detector at 3 kV.

### 2.2. Calculation of Desired Areas

**Figure 2:**
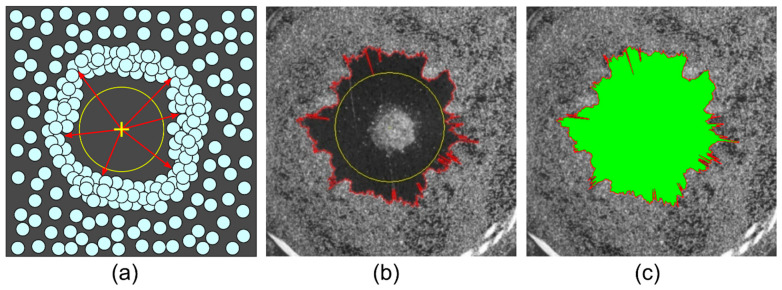
An illustration of the method for measuring the required area: (**a**) scheme of determining the boundary coordinates of the required area; (**b**) the drawing of a continuous line (a contour in red) enclosing the area; (**c**) the calculation of the desired area.

The deposit area and the area of substrate cleared from particles were measured by counting the corresponding pixels. The boundary of the area covered by particles was determined by the pixel intensity gradient in the sequence of images. The external boundary of the deposit was defined as the transition from high to low level of intensity, and the cleared area boundary—from low to high intensity, respectively. Images were processed using an in-house-developed computer program, which allowed calculating the area of the particle deposit and the area cleared from particles. The program contains a number of tools to exclude random and systematic errors in calculating the area. As an example, the procedure for the calculation of the area cleared from particles (the inner area) is schematically shown in Figure 2. Since the heater in images stands out against the substrate background, to prevent it from counting in the calculation, its area is blocked by a circle, Figure 2a. After the unknown area was calculated, the heater circle was taken into account, to avoid underestimation of the final area. A reference point (shown by a yellow cross sign) was set on the image center. Then, lines were drawn from this point (red arrows in Figure 2a) in all directions with an angular separation of 0.25° to enable measuring the boundary of the inner area in detail in images with a resolution of 1920 × 1200 pixels. The length of each line was determined by the particle closest to the heater. The contour of the inner area was interpolated using the array of boundary coordinates (Figure 2b, red outline), and then the area enclosed by that boundary was calculated (Figure 2c, indicated in green). The area bounded by the outer contour of the ring-shaped deposit, $S_{out}$, was measured in a similar way. The resulting area of the ring-shaped deposit was calculated as follows $S_{r}=S_{out}-S_{in}$.

### 2.3. Error Estimation

The error in calculating the desired area is the sum of errors in counting the pixels in the area and the random error over each series of experiments at fixed parameters. The errors in calculating the area when processing images result from the inaccuracy in determining the external boundary of the desired area. When, e.g., the radius of the area covered by particles in images takes on a value of 2 ± 0.5 pixels, the error in determining the boundary location can be estimated as 0.5 pixels. The area perimeter is calculated as the number of pixels, n, at its boundary. Therefore, taking into account the error in determining the border location, the error in determining the area will be ±n/2. The relative error in measuring the desired area in time attains a maximum value of ±1.4%. The random error was calculated over 5 measurements for each set of experimental parameters with an acceptance probability of 95%. The final values of the error in measuring the area do not exceed ±6%.

## 3. Results and Discussion

### 3.1. Mechanism of the Ring-Shaped Deposit Formation

**Figure 3:**
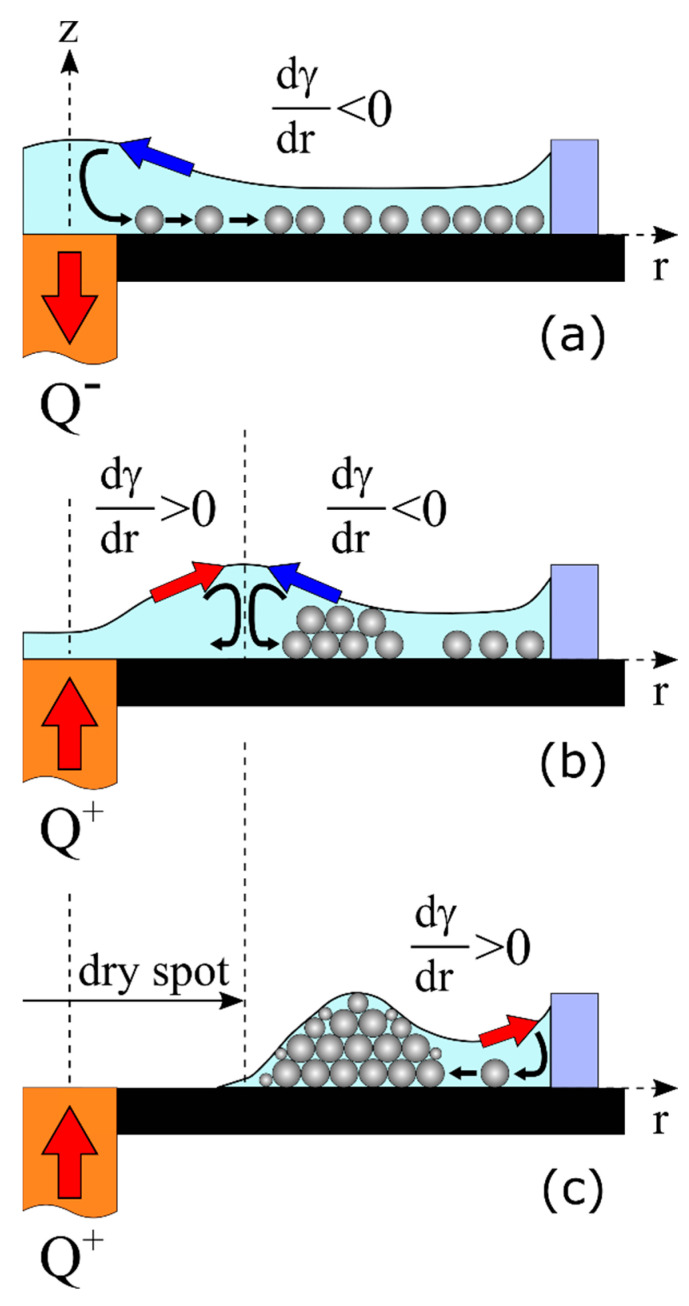
Schematic representation of the ring-shaped deposit formation. (**a**) Cooling mode (the heat is pumped out through the copper rod)—the negative surface tension gradient transfers the liquid to the center, the particles move from the center to the wall. (**b**) Heating mode—competition between negative and positive surface tension gradients, which creates counter rotating thermocapillary vortices. (**c**) Appearance of the dry spot at the central area of the cell due to thermocapillary spreading and evaporation of liquid, and formation of the outer boundary of the ring deposit due to the positive surface tension gradient.

According to the experimental procedure, the fabrication of a ring-shaped deposit (or a pattern) starts with the formation of its inner contour by applying the opposite polarity voltage to the Peltier module. In this case, the temperature of the rod decreases and, as a consequence, the liquid near the substrate flows from the rod to the wall. The basic principle of the particle transport is the following. With a local decrease in temperature of the liquid, the surface tension γ(T) locally increases according to the Guggenheim–Katayama equation.

(1) $$\gamma \left(T\right)=\gamma_{0}{\left(1-\frac{T}{T_{c}}\right)}^{n},$$

where $\gamma_{0}$ is the surface tension at the reference temperature $T_{0}$, $T_{c}$ is the critical temperature and n= 11/9 is the exponent for most organic liquids. This results in the shear stress field on the free surface of the liquid layer, which is balanced by the viscous flow of the bulk liquid

(2) $$\frac{d\gamma \left(T\right)}{dr}=\mu \frac{du}{dz}$$

where $d\gamma \left(T\right)/dr=\gamma_{T}^{\prime }\;dT/dr$ is the thermocapillary equation, $\gamma_{T}^{\prime }=-d\gamma /dT\;$ is the thermal coefficient of surface tension, dT/dr is the radial temperature gradient, μ is the viscosity of liquid, u is the horizontal component of the flow velocity, and du/dz is the vertical gradient of the flow velocity. Due to the local cooling, the temperature gradient is positive, dT/dr> 0, and that produces a negative surface tension gradient, dγ/dr< 0, hence the radially-inward thermocapillary flow along the free surface layer arises. As a result, the liquid elevates above the heat sink and forms a hill. The capillary pressure under the surface of the hill increases and causes the liquid to flow near the substrate from the heat sink. This flow is called an outward-directed bottom flow. Eventually both the flow along the free surface and the bottom flow couple and form the toroidal thermocapillary vortex in the layer. The particles sitting on the substrate exert the action of a drag force, which is caused by the viscous friction of the liquid. Under the action of the Stokes force, particles are transferred to the warm periphery and, as a result, the cooled surface becomes free from particles (Figure 3a).

**Figure 4:**
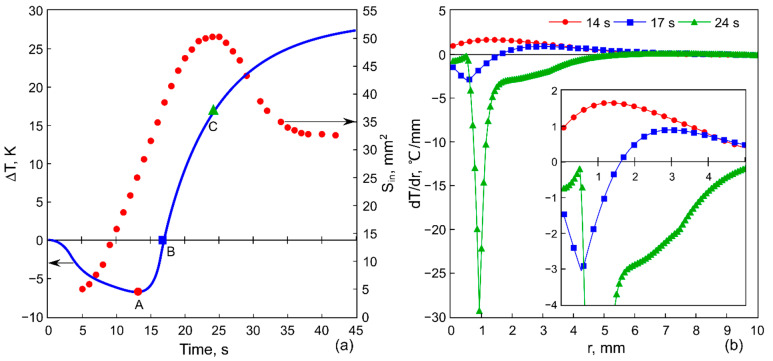
(**a**) The evolution of the inner area of the ring deposit (circle symbols) and the temperature variation on the copper rod relative to the ambient temperature (a solid line). (**b**) Radial temperature gradients at three different moments of time 14, 17 and 24 s.

To demonstrate the formation of the inner contour of the ring-shaped deposit, Figure 4a shows the evolutions of $S_{in}\left(t\right)$ and $\Delta T\left(t\right)=T\left(t\right)-T_{0}$ obtained with an electrical power P= 5 W applied to the Peltier module and a cooling time span τ= 10 s, and Figure 4b shows the radial temperature gradients dT/dr corresponding to the moments in time *t* = 14, 17 and 24 s. As can be seen in Figure 4, the inner area grows not only at the cooling stage but also during the first few seconds of heating. The reason for that effect is the following. After the cooling stage is finished, the voltage polarity on the Peltier module is changed, but due to the inertia of the heat transfer process the temperature of the rod continues to decrease slightly for another 2–3 s and reaches the minimum value ΔT≈ −7 °C for the specified power P= 5 W (Figure 4a, the point A). At this moment, the temperature gradient along the layer is positive, dT/dr> 0 (Figure 4b). This supports the negative surface tension gradient, dγ/dr< 0 responsible for the transfer of the particles away from the center and the growth of $S_{in}$ (Figure 4a). Then, in the course of heating, the temperature of the heater increases and reaches the value $T_{0}$, i.e., ΔT= 0 at t= 17 s (Figure 4a, the point B). As a result, the local temperature gradient, dT/dr< 0, is established in a zone extending from the heater center to the distance of r≅ 1.7 mm (Figure 4b, insert). In the heating zone, the surface tension decreases according to Equation (1), resulting in a positive gradient dγ/dr> 0, which leads to a change in the rotating direction of the thermocapillary vortex and the formation of a thermocapillary concave deformation (Figure 3b). At the same time, at the periphery, the positive gradient dT/dr> 0 still exists (Figure 4b); therefore, the particles continue to move away from the center increasing the area $S_{in}$. For the case depicted in Figure 4, the positive gradient dT/dr> 0 holds on near the wall of the cell for quite a long time until the temperature of the heater rises up to ΔT≈ 15 °C. Such inertia in the changing of the thermal gradient sign is attributed to the large thermal resistance at the interface between the rod ($k_{c}=$ 400 W/(m × K)—thermal conductivity) and the glass substrate ($k_{s}=$ 0.748 W/(m × K)), which delays the heat flow into the glass substrate. Therefore, the heat transfer from the heater surface at the beginning of heating is supported mainly by the convective heat transfer in the liquid. Moreover, the thermal diffusion in the glass substrate is a very long process; the diffusive relaxation time $t_{d}=R_{0}^{2}/\kappa =$ 250 s, where $R_{0}=$ 10 mm and κ≈ 4 × 10^−7^ m^2^/s is the thermal diffusivity of the glass.

**Figure 5:**
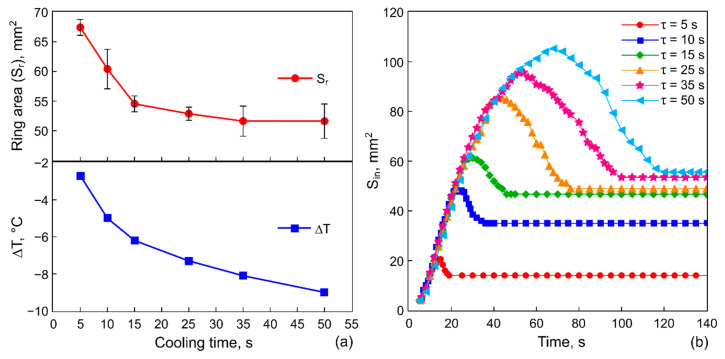
(**a**) The ring-deposit area and the temperature decreasing on the heat sink. (**b**) The evolution of the inner area (the area cleaned from particles) forming at different cooling time spans 5, 10, 15, 25, 35 and 50 s.

**Figure 6:**
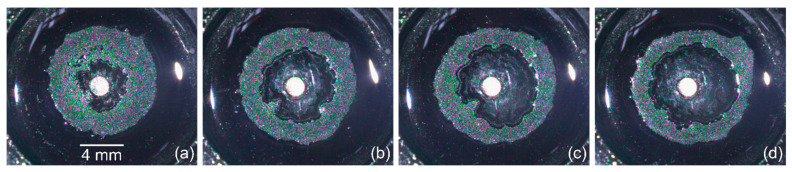
Optical images (top view) of the ring deposits of microparticles obtained at different cooling time spans and the corresponding values of ΔT on the rod for P=1.2 W: (**a**) 5 s, −3.1 °C; (**b**) 10 s, −4.7 °C; (**c**) 15 s, −6.1 °C; (**d**) 50 s, −9 °C.

**Figure 7:**
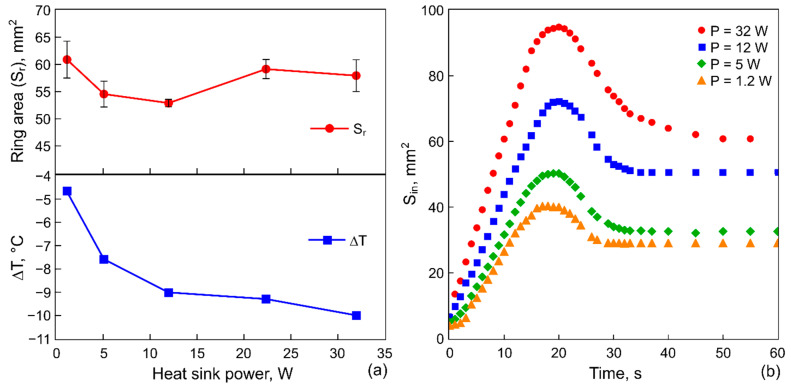
(**a**) Ring-deposit area and the temperature decrease on the rod, $\Delta T=T\left(\tau \right)-T_{0}$, versus the cooling power of the Peltier module, τ= 10 s. (**b**) Evolution of the inner area (the area cleared from particles) forming at different cooling powers of Peltier module: 1.2 W, 5 W, 12 W, and 32 W at τ= 10 s.

Thus, two opposite convective flows driven by surface tension gradients with opposite signs exist in the layer (Figure 3b) until the temperature gradient reaches a negative value, dT/dr< 0, in the entire liquid layer. In the case under consideration, this corresponds to the time t= 24 s (Figure 4b). At this moment, the outward-directed flow of the liquid and, therefore, the movement of the particles stops, and the inner area, $S_{in}$, reaches its maximum value, Figure 4a (the point C). Further, only dγ/dr> 0 is developed in the entire layer, and this leads to an inward-directed bottom flow, which transfers the particles toward the heater. This process causes a decrease in the inner area, $S_{in}$ (Figure 4a, Figure 5b and Figure 7b), and in some cases, it destroys its contour (Figure 6a). At the same time in the inner area, owing to simultaneous thermocapillary spreading and evaporation of the liquid, a dry spot appears and expands (Figure 3c). This prevents the further inward-directed transport of particles, due to a pinning of the contact liquid/solid line. As a result, the inner contour of the ring-shaped deposit attains a constant value (Figure 4a, Figure 5b and Figure 7b).

The formation of the outer boundary of the ring deposit occurs due to the action of dγ/dr> 0, which generates a flow transferring the residual particles from the periphery of the substrate to the deposit. After complete evaporation of isopropanol, the final dry ring-shaped deposit on the glass substrate is created.

### 3.2. Effects of the Cooling Power and the Cooling Time Spans on the Ring Deposit Size

Figure 5a shows the ring-shaped deposit area, $S_{r}$, and the temperature drop on the heat sink, $\Delta T=T\left(\tau \right)-T_{0}$, as functions of the cooling time spans at the constant cooling power P= 1.2 W. Figure 5b shows the evolution of the inner area, $S_{in}$, of the ring-shaped deposit for several values of the cooling time spans at P= 1.2 W.

With an increase in the cooling time span, the area of the ring deposit, $S_{r}\left(\tau \right)$, decreases, but its final inner area, $S_{in}\left(\tau \right)$, increases, which implies the formation of a multilayer structure of the particle deposition. However, a further increase in τ over 15 s (Figure 5a,b) turns out to be ineffective: both $S_{r}$ and $S_{in}$ areas change insignificantly relative to the previous values. This is related to the fact that the system is approaching thermal equilibrium, i.e., the increase in the cooling time at a given cooling power does not significantly reduce the temperature on the rod, as it is shown in Figure 5a (the temperature data). Note that a slope of the linear part of the dependency $S_{in}\left(t\right)$ is the same for all values of τ (Figure 5b). Images of the ring-shaped deposits illustrating the influence of the cooling process duration on the geometrical parameters of the ring are shown in Figure 6.

Figure 7a shows $S_{r}$ and ΔT on the heat sink as a function of the cooling power at τ= 10 s, and Figure 7b—the evolution of $S_{in}$ for different P at τ= 10 s. In contrast to the previous case, shown in Figure 5, the increase in the cooling power does not lead to a decrease in $S_{r}$, instead the area varies within the range $\Delta S_{r}\approx$ 10 mm^2^. Interestingly, the increase in the cooling power of almost three times (from 12 to 32 W) allows for the temperature to reduce by only 1.5 °C. Thus, the increase in the cooling power becomes ineffective after a certain power is reached. At the same time, $S_{in}$ and the growth rate increase with P (Figure 7b). The latter is related to the contribution of the heating in the beginning of this stage. At high powers, an extremely fast temperature rise on the rod occurs. For example, we can consider the rate of temperature rise of the rod from the minimal value reached at the cooling stage to the value at which the expansion of $S_{in}$ stops (this corresponds to the situation marked with the point C in Figure 4): it reaches 0.8 °C/s for P= 1.2 W and 5.5 °C/s for P= 32 W. Such a temperature jump gives a jump of the value and the sign of dγ/dr. As a result, the thermocapillary wave arises in the center and advances over the layer surface, sweeping particles towards the wall of the cell and increasing the inner area.

### 3.3. Morphology of the Particles Assembly in the Ring Deposit

**Figure 8:**
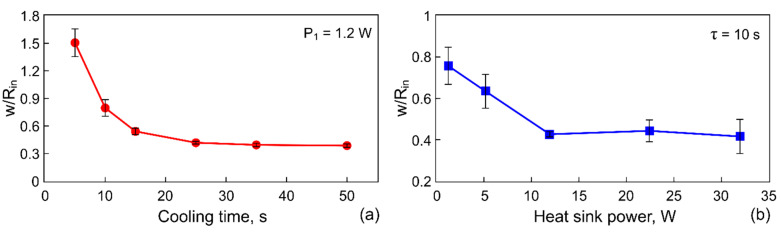
The ratio of the width of the ring to the radius of its inners area versus the cooling time spans at P= 1.2 W (**a**) and the cooling power of Peltier module at τ= 10 s (**b**).

Let us analyze the influence of the surface tension gradients, which are responsible for the formation of the ring-shaped deposit, on the particle arrangement (the distribution) over the ring width. To do this, we introduce the parameter $\bar{w}=w/R_{in}$, which represents the ratio of the ring-shaped deposit width, w, to its inner radius, $R_{in}$. Since the inner and outer boundaries of the deposit are asymmetric, we estimate averaged values as follows: $R_{in}=\sqrt{S_{in}/\pi }$, and $w=\frac{1}{\sqrt{\pi }}\left(\sqrt{S_{out}}-\sqrt{S_{in}}\right)$ using $S_{in}$ and $S_{out}$ data calculated by summing the number of pixels (see methods in Section 2.2). Figure 8 represents the dependencies of the parameter $\bar{w}=w/R_{in}$ on the cooling time span at P= 1.2 W (Figure 8a), and on the cooling power at τ= 10 s (Figure 8b).

**Figure 9:**
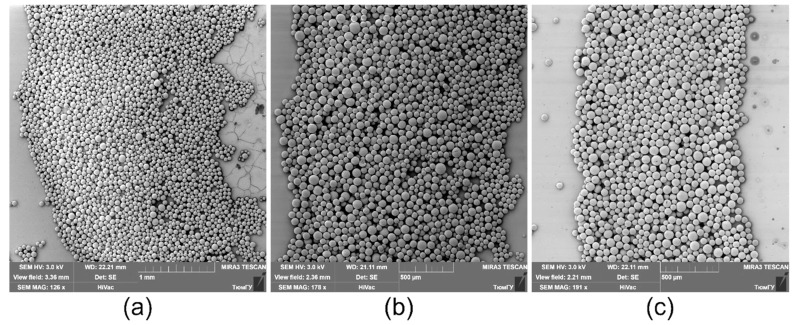
Representative arrangement (distribution) of microparticles within the width of the ring deposit for different values of τ and P: (**a**) 5 s, 5.2 W; (**b**) 5 s, 12 W; (**c**) 15 s, 32 W.

When the temperature decrease on the copper rod is |ΔT|< 5 °C (τ= 5 s, P= 1.2 W), the ring width is larger than the radius of the inner contour, $\bar{w}>1$ (Figure 6a and Figure 8a). This fact means that the ring deposit is formed mainly by the inward-directed bottom flow induced by the positive surface tension gradient dγ/dr> 0, i.e., most of the particles are transferred into the deposit from the periphery. Moreover, this flow leads to the destruction of the sharp inner boundary, which was created during the cooling stage, as it is seen Figure 6a. It is obvious that the dominance of flow caused by dγ/dr> 0 dictates the particles distribution profile along the width of the final ring-shaped deposit. Figure 9a shows a SEM image of a part of the ring where $\bar{w}>1$. It can be seen that the outer boundary is sharp and the inner one is gently-sloping and smeared. A high density of particles due to their multilayer packing is observed to be closer to the outer boundary, while along the inner boundary, particles are deposited as a monolayer.

When increasing the cooling time and decreasing the heat sink temperature, the ring width becomes smaller compared to the inner radius, $\bar{w}<$ 1 (Figure 6b,c and Figure 8). The outward-directed bottom flow due to dγ/dr< 0 (cooling stage) starts to markedly contribute to the formation of the ring-shaped deposit, which leads to a shift of the high density of the particle packing zone to the central part of the ring (Figure 9b).

For values of the cooling time spans τ> 15 s, or for values of the heat sink powers P≥ 12 W, the condition of $\bar{w}=$ const. is reached (Figure 8). In this case, the averaged inner radius of the deposit attains a maximum value $R_{in}\approx R_{0}/2$. The ring deposit is formed predominantly by transferring the particles from the center of the cell to the wall by the outward-directed bottom flow induced by dγ/dr< 0 (cooling stage). To finalize the outer boundary of the ring deposit, the particles gathered between the inner border and the wall are transferred by the flow due to dγ/dr> 0 (heating process). In this case, the particles distribute over the ring width more or less equally and the ring has both sharp edges (Figure 9c). Thus, such parameters as the inner and outer diameter, the ring width of the ring-shaped deposit as well as the particle arrangement over the ring width can be adjusted by varying the cooling time or the power applied to the Peltier module.

## 4. Conclusions

In conclusion, we have studied the process of the millimeter scale ring-shaped deposit fabrication on solid surfaces using polystyrene microparticles (microspheres) in the layer of evaporative liquid. The driving mechanism is based on the action of thermocapillary flow induced in the layer by a local thermal source/sink in the center of the surface. To manipulate the particle movement, the direction of thermocapillary flow is controlled by changing the sign of the temperature gradient in the liquid via the switching of the thermal source between heating and cooling modes. Our results show that by controlling the power applied to the heat source/sink and the duration of applied thermal flux, the rate of the ring deposit fabrication, the ring size and the particle arrangement over the width of the ring can be tuned. The proposed method enables the simultaneous control of the millimeter scale and microscale processes of particle transfer and arrangements and can be used for creating structures of nano- and microparticles on spatial mesoscales.

## Data Availability

All data are available from the authors.
